# Intracellular *Porphyromonas gingivalis* Promotes the Tumorigenic Behavior of Pancreatic Carcinoma Cells

**DOI:** 10.3390/cancers12082331

**Published:** 2020-08-18

**Authors:** JebaMercy Gnanasekaran, Adi Binder Gallimidi, Elias Saba, Karthikeyan Pandi, Luba Eli Berchoer, Esther Hermano, Sarah Angabo, Hasna′a Makkawi, Arin Khashan, Alaa Daoud, Michael Elkin, Gabriel Nussbaum

**Affiliations:** 1 The Institute of Dental Sciences, Hebrew University, Hadassah Faculty of Dental Medicine, Jerusalem 9112102, Israel; jebmercy@gmail.com (J.G.); adi.binder@mail.huji.ac.il (A.B.G.); elias.saba@mail.huji.ac.il (E.S.); karthike.pandi@mail.huji.ac.il (K.P.); lubae@ekmd.huji.ac.il (L.E.B.); Sarah.angabo@mail.huji.ac.il (S.A.); hasnaa.makkawi@mail.huji.ac.il (H.M.); arin.khashan@mail.huji.ac.il (A.K.); alaa.daoud@mail.huji.ac.il (A.D.); 2 Sharett Oncology Institute, Hadassah-Hebrew University Medical Center, Jerusalem 9112102, Israel; estherhermano@gmail.com

**Keywords:** *Porphyromonas gingivalis*, periodontitis, pancreatic neoplasms, tumor hypoxia, carcinogenesis

## Abstract

*Porphyromonas gingivalis* is a member of the dysbiotic oral microbiome associated with oral inflammation and periodontal disease. Intriguingly, epidemiological studies link *P. gingivalis* to an increased risk of pancreatic cancer. Given that oral bacteria are detected in human pancreatic cancer, and both mouse and human pancreata harbor microbiota, we explored the involvement of *P. gingivalis* in pancreatic tumorigenesis using cell lines and a xenograft model. Live *P. gingivalis* induced proliferation of pancreatic cancer cells; however, surprisingly, this effect was independent of Toll-like receptor 2, the innate immune receptor that is engaged in response to *P. gingivalis* on other cancer and immune cells, and is required for *P. gingivalis* to induce alveolar bone resorption. Instead, we found that *P. gingivalis* survives inside pancreatic cancer cells, a trait that can be enhanced in vitro and is increased by hypoxia, a central characteristic of pancreatic cancer. Increased tumor cell proliferation was related to the degree of intracellular persistence, and infection of tumor cells with *P. gingivalis* led to enhanced growth in vivo. To the best of our knowledge, this study is the first to demonstrate the direct effect of exposure to *P. gingivalis* on the tumorigenic behavior of pancreatic cancer cell lines. Our findings shed light on potential mechanisms underlying the pancreatic cancer–periodontitis link.

## 1. Introduction

Pancreatic ductal adenocarcinoma (PDAC) is among the most aggressive and least treatable forms of cancer [1]. PDAC develops in an inflammatory environment that is present even in low-grade premalignant lesions from individuals without a history of acute or chronic pancreatitis [2]. Several studies demonstrate an association between PDAC and periodontitis, a common and chronic inflammatory disease of the oral cavity that is driven by a dysbiotic microbiome [3,4,5]. Periodontal disease may influence pancreatic cancer by elevating inflammatory mediators that promote tumor development, or by direct effects of bacteria on tumor cells or their microenvironment. A large, prospective, population based-study demonstrated a highly significant association between carriage of *Porphyromonas gingivalis* (*P. gingivalis*), and the risk of pancreatic cancer development [4]. *P. gingivalis* is a gram negative, anaerobic bacteria that thrives in the inflamed environment of periodontal lesions, and escapes the bactericidal activity of innate immune cells by activating Toll-like receptor 2 (TLR2) signaling through PI3K [6,7]. *P. gingivalis* is linked to several extra-oral disorders, and migrates to tissues remote from the oral cavity [8,9,10,11,12,13]. Oral bacteria are found in PDAC samples, and both mouse and human pancreata harbor microbiota [14,15,16,17], although how oral pathogens reach and invade pancreatic tissue has not been addressed. Hematogenous spread is a likely route of access for oral pathogens since transient bacteremia follows daily oral hygiene procedures, especially in individuals with periodontitis who harbor high levels of periodontal pathogens [18]. Importantly, *P. gingivalis* carriage in the oral cavity is correlated with pancreatic cancer risk even when present years prior to pancreatic cancer diagnosis [4], consistent with the notion that migration of *P. gingivalis* to the pancreas from the oral cavity, even if transient and intermittent, increases cancer risk.

Despite the epidemiological evidence implicating *P. gingivalis* in pancreatic tumorigenesis [19], the direct effects of this bacterium on PDAC tumor progression have not been investigated, and the mechanisms underlying the *P. gingivalis*–PDAC link remain unclear. Here, we provide the first evidence that *P. gingivalis* exerts direct pro-tumorigenic effects on pancreatic cancer cells. Although PDAC cells are reported to express some Toll-like receptors [20], and *P. gingivalis* induces proliferation of oral epithelial tumor cells in a TLR2-dependent manner [21], we found that pancreatic tumor cell proliferation is enhanced by *P. gingivalis* independently of TLR2. Since *P. gingivalis* is known to invade and persist in epithelial cells [22,23], we next addressed the hypothesis that *P. gingivalis* intracellular survival is linked to its ability to promote pancreatic tumor cell proliferation. Importantly, we found that hypoxia, a dominant feature of the PDAC microenvironment [24,25], greatly enhances *P. gingivalis* intracellular survival. To the best of our knowledge, our study is the first to test the mechanistic involvement of *P. gingivalis* in pancreatic tumorigenesis, applying in vitro tools and a xenograft pancreatic carcinoma model in vivo. Our results reveal a previously unknown direct effect of *P. gingivalis* on PDAC progression, highlighting the importance of the interplay between hypoxia and *P. gingivalis* intracellular survival in this process.

## 2. Results

### 2.1. P. gingivalis Infection Enhances Pancreatic Tumor Cell Proliferation

**Figure 1:**
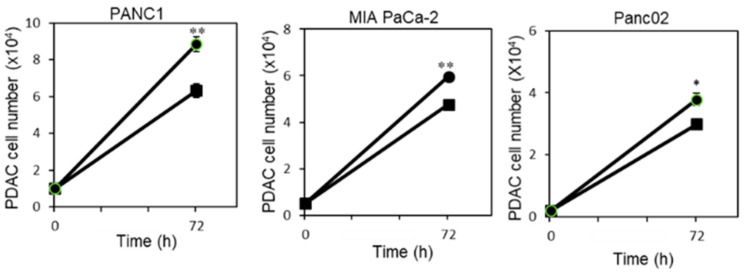
*P. gingivalis* infection enhances PDAC cell proliferation. PDAC cells (PANC1, MIA PaCa-2, and Panc02) were cultured in the absence of antibiotics alone or in the presence of *P. gingivalis* (MOI 10 bacteria per cell, 4 wells per condition) and cell number was calculated at the indicated time points (squares indicate cells without bacteria, and circles indicate cells with bacteria). One representative experiment of three is shown. Two-tailed *t*-test was performed for statistical analysis. * *p* ≤ 0.05, ** *p* ≤ 0.01.

To investigate how *P. gingivalis* influences PDAC progression, we first compared the proliferation of pancreatic carcinoma cell lines in the absence or presence of *P. gingivalis*. As shown in Figure 1, *P. gingivalis* infection significantly increased the proliferation rate of the human PDAC cell lines PANC1 and MIA PaCa-2, and the mouse cell line Panc02.

*P. gingivalis* was previously shown to promote growth of cancer cells (of non-pancreatic origin, i.e., oral squamous cell carcinoma) through a TLR2 dependent mechanism [21]. Therefore, we next hypothesized that TLR2 is involved in mediating increased proliferation of PDAC cells in the presence of *P. gingivalis*. However, blocking TLR2 with a TLR2-neutralizing antibody (clone T2.5, [6]) did not diminish the *P. gingivalis*-induced proliferation of PDAC cells (Figure S1 (Supplementary Materials)). Moreover, RT-PCR analysis showed that PDAC cell lines used in our experiments do not express TLR2, in line with previous reports [26]. Accordingly, we were unable to detect induction of inflammatory cytokines typically associated with TLR2 activation in the PDAC cell lines following exposure to *P. gingivalis* (e.g., IL-6 and TNFα), further confirming that the growth-promoting effects of *P. gingivalis* on pancreatic carcinoma cells are not mediated by TLR2.

### 2.2. Enhanced Tumor Cell Proliferation Correlates with P. gingivalis Intracellular Survival

**Figure 2:**
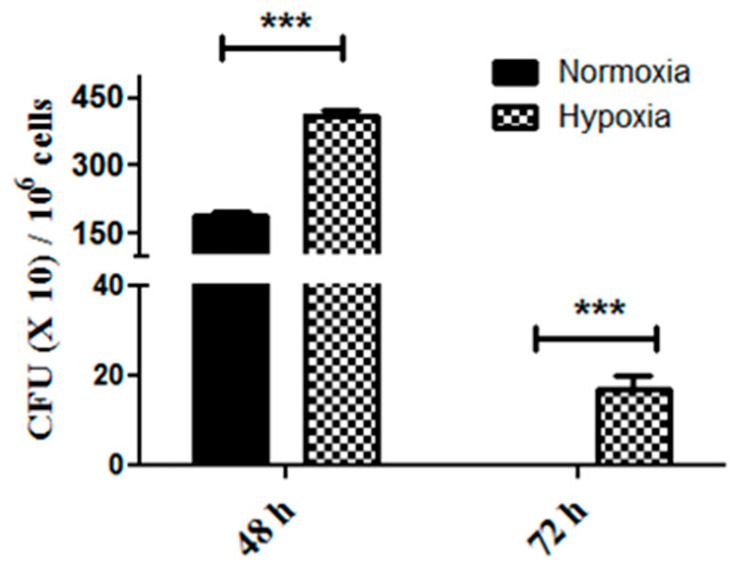
*P. gingivalis* intracellular survival assay. PANC1 cells were infected with *P. gingivalis* for 1 h in normoxic vs. hypoxic conditions and treated with antibiotics to remove all extracellular bacteria. Cells were lysed at the indicated time points, and lysates were plated on anaerobic blood agar plates to determine CFU. The experiment was performed three times. Data show the mean ± SD of a representative experiment (*n* = 3). Two-tailed *t*-test was performed for statistical analysis. *** *p* ≤ 0.005.

*P. gingivalis* is known to survive and even multiply within gingival epithelial cells [23,27]. We next hypothesized that proliferation induced by *P. gingivalis* is a consequence of intracellular survival. To study intracellular survival, we infected pancreatic cancer cells for 1 h, washed and treated the cells with antibiotics to kill extracellular bacteria, and then incubated the cells for extended periods of time. Under these conditions, no live bacteria were recoverable from the culture media collected after antibiotic treatment, in the presence or absence of the cancer cells. We recovered live organisms from within pancreatic cancer cells two days after infection; however, no live bacteria were recovered 3 days after infection (Figure 2). Incubation of pancreatic cancer cells in 1% oxygen to mimic the hypoxic conditions of pancreatic tumors greatly increased *P. gingivalis* intracellular survival and persistence, demonstrated by the difference in the number of colonies recovered at 48 h in the different conditions, and the fact that live *P. gingivalis* was recovered from the cancer cells grown for 72 h in hypoxic conditions, but not from those cells infected with the same *P. gingivalis* and then grown in normoxic conditions (Figure 2). Therefore, *P. gingivalis* is slowly cleared from inside PDAC cells in a manner related to environmental oxygen levels.

**Figure 3:**
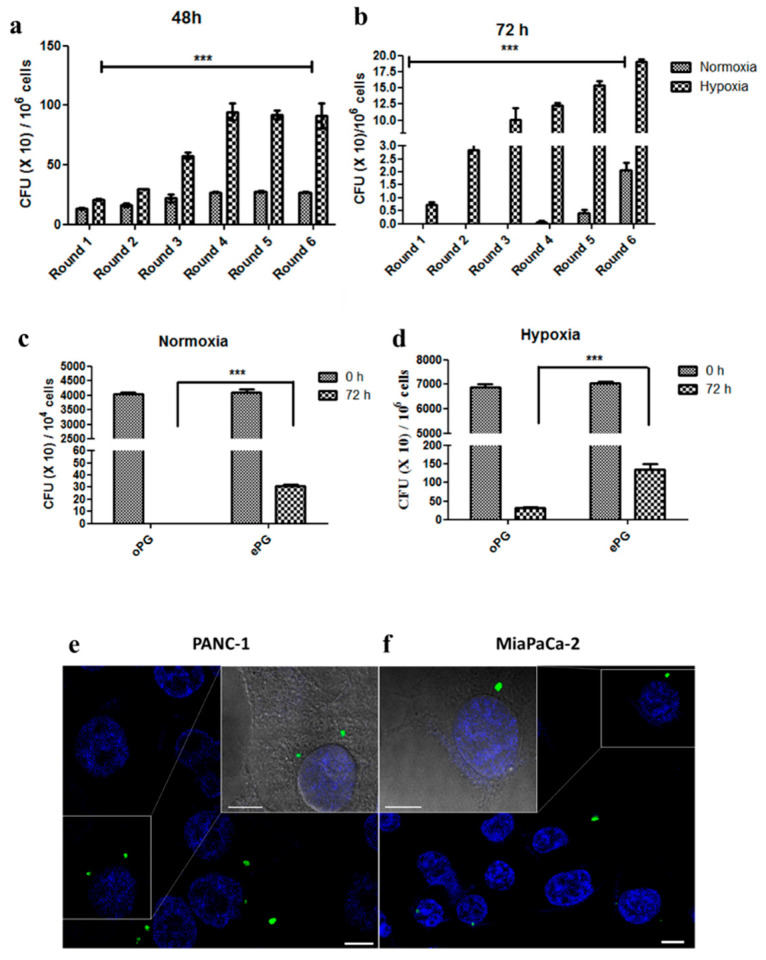
*P. gingivalis* intracellular survival in PDAC cells can be enhanced in vitro. (**a**,**b**) Intracellular *P. gingivalis* CFU recovered 48 h and 72 h after infection. (**c**,**d**) Intracellular “original” vs. “enhanced” *P. gingivalis* (see text for explanation) recovered immediately after antibiotic treatment (0 h) or after 72 h in normoxic or hypoxic conditions. Experiment was performed three times. Data show the mean ± SD of a representative experiment (*n* = 2 wells per condition). Two-way ANOVA was performed for statistical analysis. *** *p* ≤ 0.005. (**e**,**f**) FITC-labeled *P. gingivalis* was used to infect PANC1 or Mia Paca-2 cells. After 24 h infection, cells were fixed and nuclei were stained with DAPI prior to visualization using a NIKON confocal microscope at 60× magnification. One cell is magnified and shown with brightfield overlay for each image. The experiment was performed twice.

To explore the relationship between intracellular survival and the effect of *P. gingivalis* on proliferation, we endeavored to enhance the intracellular survival of *P. gingivalis* strain 381 by successive rounds of infection in either normoxia or hypoxia. For PANC1 cells grown in normoxia, a single *P. gingivalis* colony recovered from inside cells that were lysed at 48 h was expanded and used to infect cells in the next round. After four rounds of infection, we were now able to recover *P. gingivalis* from cells grown for 72 h in normoxic conditions, and the number of intracellular *P. gingivalis* colonies continued to increase with successive rounds of infection (Figure 3a). For hypoxic conditions, a single *P. gingivalis* colony recovered from inside cells that were lysed at 72 h was expanded and used to infect cells in the next round. Six rounds of infection (each time using a colony recovered from PANC1 cells grown for 72 h to re-infect fresh PANC1 cells) increased the number of intracellular *P. gingivalis* colonies recovered at 72 h in hypoxic conditions greater than 20-fold (Figure 3b). To confirm that the bacteria were adapted to intracellular survival rather than enhanced invasiveness, we infected fresh PANC1 cells for 1 h with the original laboratory stock of *P. gingivalis* (“oPG”) vs. a *P. gingivalis* stock based on bacteria that had been enhanced for intracellular survival by six successive rounds of infection and recovery from inside the PANC1 cells (we refer to this as “ePG”). The PANC1 cells were treated with antibiotics to eliminate extracellular bacteria, and then cells were immediately lysed with water to recover the intracellular bacteria (“0 h”), vs. incubation for 72 h and then lysis. As expected, there were large differences in the number of CFU recovered at 72 h, however there were no differences in the number of bacteria recovered immediately after infection (the 0 h time point), demonstrating that the bacteria (oPG vs. ePG) entered the cells to the same degree but differed in their ability to persist inside the cells (Figure 3c,d). Consistent with the intracellular survival, fluorescently-labeled ePG was detected by confocal microscopy in both PANC1 and MIA PaCa-2 cells (Figure 3e,f).

**Figure 4:**
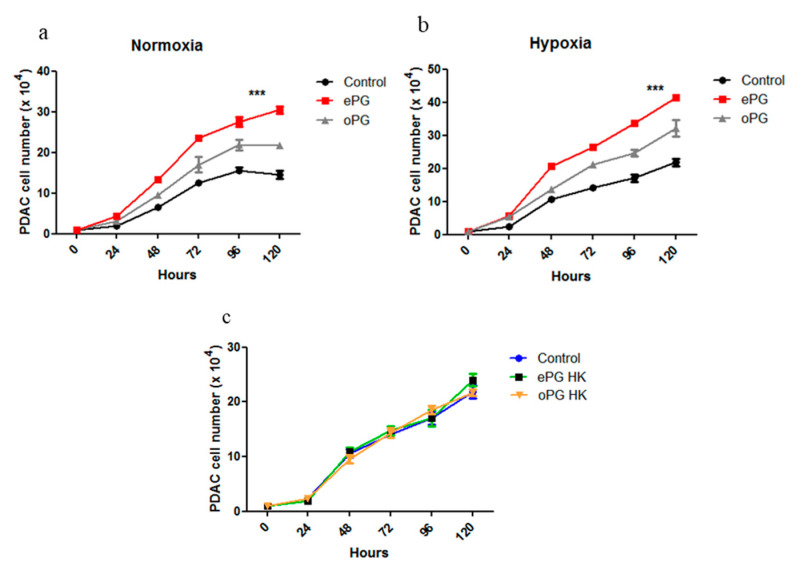
Enhanced *P. gingivalis* intracellular survival correlates with increased PDAC proliferation. (**a**) PANC1 cells were cultured in normoxia or (**b**) hypoxia alone or in the presence of oPG vs. ePG (MOI 10). For each of the conditions (normoxia vs. hypoxia), the ePG generated in those conditions was used. Extracellular bacteria were killed with antibiotics after one hour of infection, and cell number was calculated at the indicated time points. (**c**) Cells were cultured in hypoxic conditions alone (control) or in the presence of heat-killed (HK) ePG or oPG, and cell number was determined at the indicated time points. Experiment was performed three times. Data show the mean ± SD of a representative experiment (*n* = 3). Two-way ANOVA was performed for statistical analysis. *** *p* ≤ 0.005.

We next compared the effect of oPG vs. ePG on the proliferation of PANC1 cells, in either normoxia or hypoxia. In both conditions, *P. gingivalis* adapted for intracellular survival enhanced proliferation significantly more than the original stock of bacteria (Figure 4a,b). Exposure to heat-killed ePG did not increase cell proliferation, demonstrating that live bacteria are required to enhance proliferation (Figure 4c, similar results obtained for oPG).

**Figure 5:**
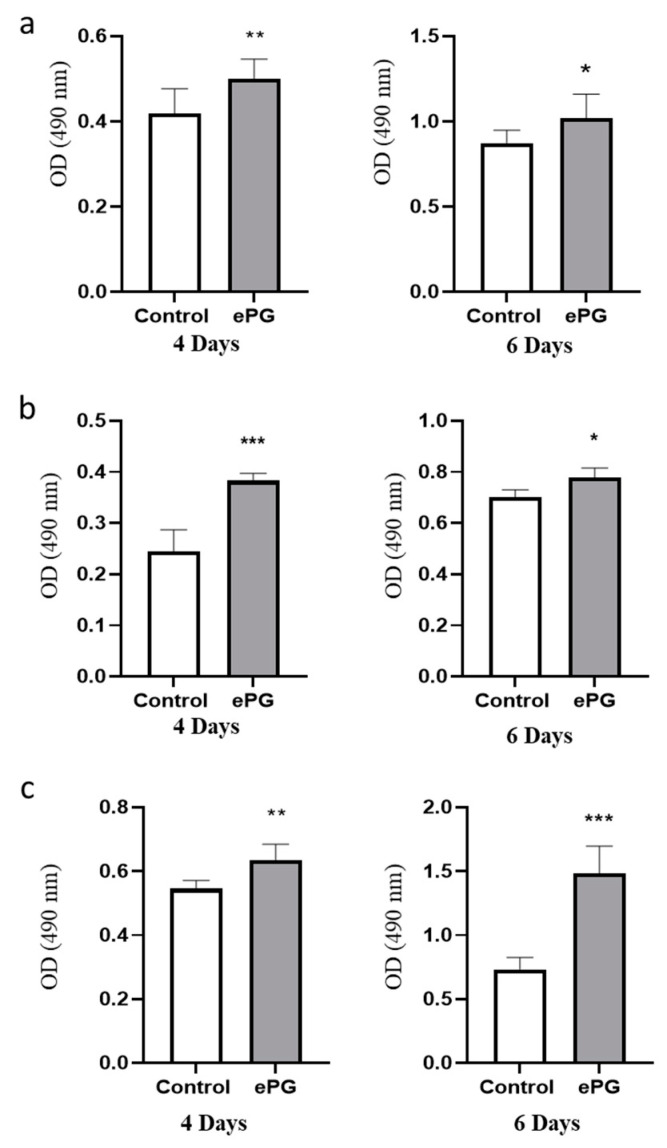
Enhanced proliferation induced by ePG in three human PDAC cell lines. (**a**) PANC1 cells, (**b**) MIA PaCa-2, and (**c**) BxPC-3 cells were cultured in hypoxia alone or with ePG (MOI 10). Extracellular bacteria were killed with antibiotics after one hour of infection, and cell proliferation was determined by MTS after 4 or 6 days. Two-tailed *t*-test was performed for statistical analysis. * *p* ≤ 0.05, ** *p* ≤ 0.01, *** *p* ≤ 0.005.

The enhanced proliferation induced by ePG was confirmed in three PDAC cell lines at four and six days after infection using an additional test of cell viability (Figure 5a–c). In agreement with increased proliferation, we also found that *P. gingivalis*-infected cells had elevated levels of cyclin D1, an important regulator of proliferation/cell cycle progression in a variety of tumor types, including PDAC (Figure S2 (Supplementary Materials)) [28]. In addition, we noted that *P. gingivalis* infection resulted in increased levels of heparanase, an enzyme involved in PDAC tumor growth and aggressiveness (Figure S2 (Supplementary Materials)) [29,30].

### 2.3. P. gingivalis Enhances Tumor Growth and Akt Activation

**Figure 6:**
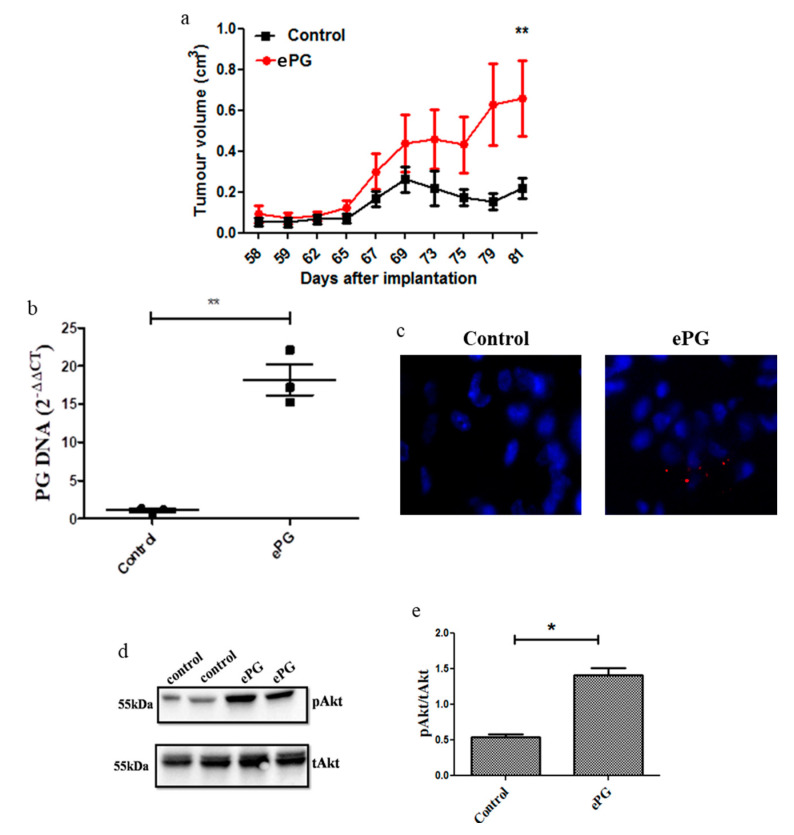
*P. gingivalis* enhances tumor growth and Akt phosphorylation. (**a**) PANC1 cells were incubated in hypoxia and infected with ePG or sham infected, and then injected subcutaneously to SCID mice (*n* = 7 per group). From the time tumors were palpable and through 11 weeks, tumor volume was monitored daily (in the control group one mouse did not develop a tumor and was not included in the analysis). (**b**) Following sacrifice on day 81 post implantation, tumors (*n* = 3 per group) were frozen and DNA was extracted as per the methods. RT PCR was performed using primers for the 16s *P. gingivalis* rDNA gene and the human 18S gene. Relative expression was calculated using the 2-ΔΔCT method. ** *p* = 0.0011. (**c**) For each group, three tumors were fixed in 4% paraformaldehyde and then embedded in OCT. Frozen sections were stained with a Cy3-labeled oligonucleotide specific for the *P. gingivalis* 16s rRNA gene (red), and nuclei were counterstained with DAPI (blue). Representative images of one control and one *P. gingivalis*-infected tumor at 20× magnification are shown. (**d**,**e**) Tumor protein lysates from two mice in each group were analyzed by Western blot for Akt phosphorylation (**d**) and protein levels were determined by densitometry (**e**). * *p* ≤ 0.05, ** *p* ≤ 0.01.

To investigate whether *P. gingivalis* influences pancreatic tumor growth in vivo, we injected severe combined immunodeficient (SCID) mice subcutaneously with PANC1 cells that were infected with ePG prior to injection, and control mice were injected with uninfected PANC1 cells. Cells were not treated with antibiotics due to a concern that antibiotic treatment would interfere with tumor engraftment; however, cells were extensively washed, and no live *P. gingivalis* was recovered from the extracellular medium prior to implantation. All mice injected with *P. gingivalis*-infected PANC1 cells (*n* = 7) developed xenograft tumors vs. six of seven mice injected with uninfected PANC1 cells. Consistent with the in vitro results, xenograft tumors formed by *P. gingivalis*-infected PANC1 cells were significantly larger than tumors formed by control cells (Figure 6a). *P. gingivalis* DNA was still detectable in tumor tissue at the time of sacrifice (Figure 6b), and small numbers of *P. gingivalis* were visible by fluorescence in-situ hybridization [31] 11 weeks after implantation (Figure 6c). As *P. gingivalis* has previously been shown to induce PI3K/Akt signaling in host cells of non-cancerous origin (i.e., gingival epithelial cells [32,33]), and given the key role of the PI3K/Akt pathway in PDAC progression [34], we next examined Akt signaling in the two groups of tumors. Akt signaling was strongly upregulated in tumors derived from *P. gingivalis*-infected cells compared to control tumors as evident by increased phospho-Akt (pAkt) levels (Figure 6d,e). These in vivo findings were in agreement with the in vitro induction of pAKT by *P. gingivalis* infection of the PDAC cell lines (Figure S3 (Supplementary Materials)). Of note, Akt phosphorylation was persistently induced by infection of PANC1 cells, and was still significantly elevated compared to baseline at 24 h post challenge, and a similar effect was observed by infection of an additional pancreatic cancer cell line, MIA PaCa-2 (Figure S4 (Supplementary Materials)). Collectively, these findings suggest that the pro-tumorigenic effects of *P. gingivalis* may be explained, in part, by activation of the Akt signaling cascade.

## 3. Discussion

The prognosis of pancreatic ductal adenocarcinoma is dismal, and most cases are diagnosed at late stages of disease in individuals without known risk factors [35]. Therefore, there is an urgent need to identify new, modifiable risk factors that contribute to tumor development and aggressiveness. Prospective epidemiologic studies have linked the periodontal bacterium *P. gingivalis* to pancreatic cancer [4,5]; however, functional studies that explain this link are lacking. In this study, we demonstrated that *P. gingivalis* directly affects PDAC cells. *P. gingivalis* induces PDAC cell proliferation, which is enhanced in hypoxic conditions (characteristic for pancreatic carcinoma [24]). Promotion of proliferation is linked to the intracellular survival of the bacteria (Figure 3) and its ability to augment Akt signaling and cyclin D1 expression (Figure 6 and Figures S2–S4 (Supplementary Materials)), one of the crucial pathways implicated in PDAC progression [28,34].

TLR2 plays a major role in the host response to *P. gingivalis*; however, the bacteria manipulate TLR2 signaling in immune cells to evade bactericidal activity while inducing inflammation [6,7,36]. *P. gingivalis* also promotes proliferation of oral carcinoma cells in a TLR2-dependent manner [21]. Nevertheless, here we show that in the setting of pancreatic cancer, tumor-promoting effects of *P. gingivalis* are TLR2-independent, and enhanced by increasing the ability of bacteria to survive within the cells. Intracellular *P. gingivalis* survival is a well-known phenomenon in gingival epithelial cells [37], and *P. gingivalis* has been shown to multiply and even spread between gingival epithelial cells during the first day after infection, although over time, in culture, *P. gingivalis* persistence in gingival epithelial cells decreases [27,38]. Intracellular survival in cancer cells is a novel mechanism whereby bacteria may influence cancer development. Bacterial adaptation to intracellular life in cancer cells, and the kinetics of intracellular persistence, may differ from infection of gingival epithelial cells [23,27,37]. We found that hypoxia further increases *P. gingivalis* intracellular survival and the proliferation of PDAC cells. In contrast, hypoxia upregulates the bactericidal activity of keratinocytes against Gram-positive bacteria [39]. Since hypoxia is a prominent feature of pancreatic tumors [24], and an important determinant of PDAC malignancy [24,25], pancreatic tumors may be an ideal niche for *P. gingivalis* intracellular survival. Our findings provide the first evidence for a direct effect of intracellular *P. gingivalis* on activation of cancer signaling pathways (evidenced by prolonged Akt phosphorylation in vitro, and in the xenograft tumors); however, the full spectrum of cancer properties affected by *P. gingivalis* infection in PDAC, and the precise molecular mechanisms underlying the effects have still to be determined. For example, in healthy epithelial cells, *P. gingivalis* or its surface components have been shown to both induce and inhibit expression of apoptotic factors and cellular apoptosis [40,41,42,43,44]. Our ongoing studies are focused on how intracellular *P. gingivalis* affects the PDAC apoptosis machinery, especially since BCL2, a powerful anti-apoptotic factor whose expression correlates with apoptotic resistance and metastatic potential in PDAC [45], can either be increased or inhibited by *P. gingivalis* [43,44]. One intriguing possibility is that intracellular *P. gingivalis* may influence tumor development by synergizing with other oncogenic factors that upregulate the same pathways, such as mutant KRAS, the hallmark genetic mutation of PDAC [46]. In the context of PDAC development, it is important to explore the potential synergy between intratumoral bacteria and oncogenic signaling pathways.

*P. gingivalis* strains available from the ATCC were isolated from humans with periodontitis decades ago, and have been passaged enumerable times in culture which may lead to loss of virulence. Furthermore, virulence mechanisms, including intracellular survival, vary among available laboratory strains [47], suggesting that clinical isolates may differ in their ability to persist in cancer cells. Starting with the laboratory strain *P. gingivalis* 381 isolated by Tanner in 1979 [48], we demonstrated that intracellular persistence can be enhanced in vitro, suggesting that it may occur in vivo as well. It will be critical to determine the changes bacteria undergo to adapt to intracellular life in cancer cells, and if clinical isolates of *P. gingivalis* differ in their potential to influence pancreatic tumorigenesis.

A convergence of human and mouse studies demonstrate that oral cavity and intestinal microbes (including *P. gingivalis*) can colonize the healthy and tumor-containing pancreas [12,14,15,16,17,49,50]. *P. gingivalis* is a member of the phylum Bacteroidetes, which was among the most abundant phyla in human PDAC samples in one study [15], although it was much less abundant in other studies [14,16]. Quantitative assessment of the pancreatic microbiota at a single time point may be inadequate to reveal a role for particular microbes in cancer progression. The prospective study by Fan et al. correlated salivary *P. gingivalis* with pancreatic cancer risk even when at least two years separated the two events [4]. Therefore, *P. gingivalis* can be a transient traveler to the pancreas during the critical time course of PDAC maturation, even if it is not a significant member of the pancreatic microbiota at the time of diagnosis. Although a limitation of the present study is that a single model was used for in vivo confirmation, our report on a new role of intracellular *P. gingivalis* in PDAC may help to disentangle the enormous complexity of the PDAC–periodontitis link.

## 4. Materials and Methods

### 4.1. Bacteria and Growth Conditions

*P. gingivalis* strain 381 or 33,277 were cultured in Wilkins broth (Oxoid, Basingstoke, UK) under anaerobic conditions (85% N_2_, 5% H_2_ and 10% CO_2_) in AnaeroJar^TM^ at 37 °C. Optical density at 650 nm of 0.1 was determined to be equivalent to 10^10^ bacterial counts per mL.

### 4.2. Cell Lines and Culture Conditions

Human pancreatic carcinoma cell lines MIA PaCa-2 and PANC1 (authenticated by STR profiling at the Genomics Center of the Biomedical Core Facility, Technion University, Haifa, Israel), and the mouse pancreatic carcinoma cell line Panc02 [51] were maintained in DMEM (Sigma-Aldrich, Rehovot, Israel) supplemented with 10% fetal calf serum, 2 mM L-glutamine, penicillin (100 units/mL), and streptomycin (100 µg/mL; Biological Industries, Beit Haemek, Israel). The cells were cultured at 37 °C and 5% CO_2_ (normoxia). To induce a hypoxic environment, cells were cultured at 37 °C in a Hypoxia Incubator Chamber (STEMCELL Technologies, Vancouver, BC, Canada) flushed with a gas mixture of 5% CO_2_/94% N_2_/1% O_2_ for 4 h prior to infection with *P. gingivalis*.

### 4.3. Cell Proliferation Assay

Cells were plated in 24-well plates at 10^4^ cells per well in DMEM. One hour prior to bacterial infection, the medium was changed to DMEM without antibiotics and cells were incubated with *P. gingivalis* (MOI 10), or left untreated. Cell numbers were counted at the indicated time intervals using a hemocytometer. Alternatively, cell proliferation was measured using the Promega CellTiter 96^®^ AQueous One Solution Cell Proliferation Assay (MTS, Cat No. G5430, Promega, city, Madison, WI, USA). We plated 1000–2000 pancreatic cancer cells/well in 96 well plates, and the cells were incubated in hypoxia for 4 h before infection with *P. gingivalis* MOI 10 for 1 h. Cells were then washed, treated with metronidazole and gemtamycin for 1 h, and incubated for 4 or 6 days in hypoxia. MTS solution (20 μL) was added to each well and incubated at 37 °C for 1.5 h. The absorbance at 490 nm was used to evaluate cell proliferation.

### 4.4. P. gingivalis Intracellular Survival Assay (ICS)

Approximately 0.8 to 1 × 10^6^ PANC1 cells per well were plated in 6-well plates and infected with *P. gingivalis* at multiplicity of infection (MOI) 10 in duplicate or triplicate for 1 h in either normoxic, or hypoxic conditions in the absence of antibiotics. For hypoxia, cells were pre-incubated in hypoxic conditions for 4 h, and then infection and subsequent steps were performed in hypoxic conditions. For both normoxic and hypoxic conditions, cells were washed twice after infection, and then treated with metronidazole (0.22 mg/mL) and gentamycin (0.3 mg/mL) for an hour to eradicate all remaining extracellular adherent bacteria. After this treatment, cells were washed twice and incubated in full medium (as above, i.e., supplemented with penicillin and streptomycin, but without metronidazole and gentamicin) for 24, 48 and 72 h in either normoxia or hypoxia. Cells were then lysed by treating with ice-cold distilled water for 20 min. Serial dilutions of cell lysates were plated on anaerobic blood agar plates (Novamed, Jerusalem, Israel), and incubated under anaerobic conditions for a week to determine the colony forming units (CFU) in each condition. To enhance *P. gingivalis* intracellular survival, one *P. gingivalis* colony recovered in each condition (normoxia vs. hypoxia) was expanded in liquid medium, and then used to infect fresh PANC1 cells. This was performed successively, and each infection–recovery cycle is referred to as a “round” of infection.

### 4.5. Detection of Intracellular P. gingivalis

We plated 30,000 cells/well in IBIDI chamber slides (Ibidi, Martinsried, Germany), and incubated in hypoxia for 4 h prior to infection with FITC-labeled *P. gingivalis* MOI 10 for 1 h. *P. gingivalis* was labeled with 0.1 mg/mL FITC (Sigma) in carbonate buffer (pH 9.5) for 20 min at RT and then extensively washed in PBS. Infection and all subsequent steps were carried out in hypoxia. Following infection, cells were washed, treated with metronidazole and gentamicin for 1 h, washed, and incubated for 24 h. The wells were then fixed with 2% formaldehyde for 15 min and blocked with 0.1% Triton/2% BSA. Nuclei were stained with DAPI 3 μL/mL blocking solution for 30 m. Cells were washed twice, and then IBIDI mounting medium was applied. Multi-projection images were obtained using a NIKON confocal fluorescent microscope at 60× magnification.

### 4.6. Analysis of Gene Expression by qRT-PCR

Total RNA was isolated from PANC-1 cells using TRIzol (Invitrogen, Thermo Fisher Scientific, Madison, WI, USA), according to the manufacturer’s instructions, and quantified by spectrophotometry (NanoDrop One, Thermo Fisher Scientific). After oligo (dT)-primed reverse transcription of 1 μg total RNA, the resulting single stranded cDNA was amplified using the primers listed below. Real-time quantitative PCR (qRT-PCR) analysis was performed on a BioRad CFX Connect system (Bio-Rad, Hercules, CA, USA). The PCR reaction mix (10 µL) was composed of 5 µL QPCR sybr master mix (Bio-Rad), 1 µL cDNA, and a final concentration of 0.5 µM of each primer diluted in ultra-pure water. Glyceraldehyde 3-phosphate dehydrogenase (GAPDH) was used as an internal control for normalization. The following primers were utilized: Human GAPDH F: 5′-TCCACTGGGGTCTTGACG-3′, R: 5′-GGCAGAGATGATGACCCTTTT-3′. Human Cyclin D1 F: 5′-TGTTCGTGGCCTCTAAGATGAAG-3′, R: 5′-AGGTTCCACTTGAGCTTGTTCAC-3′. Human Heparanase F: 5′-GTTCTAATGCTCAGTTGCTCCT-3′, R: 5′-ACTGCGACCCATTGATGAAA-3′.

### 4.7. Tumor Growth In Vivo

Ten to twelve-week-old SCID mice were obtained from Envigo (Jerusalem, Israel) and housed in the Specific Pathogen-Free (SPF) facility of the Hebrew University. PANC1 cells were incubated in hypoxic conditions for four hours prior to infection with *P. gingivalis* enriched for intracellular survival (MOI of 10) vs. sham infection for one hour in hypoxia. Cells were washed three times and five million cells in 200 μL PBS per mouse were implanted into the subcutaneous space of the flank (*n* = 7). Mice were monitored daily and tumor length and width was calculated using a caliper from the first day that the tumor was palpable. Tumor volumes based on caliper measurements were calculated by the modified ellipsoidal formula (tumor volume = (length × width^2^)/2). Animal experiments were approved by the Institutional Animal Care and Use Committee using protocol MD-13-12544.

### 4.8. Detection of P. gingivalis in Tumor Tissue

Intact tumor tissue was isolated from mice at the end of the experiment (day 81) and DNA from tumor samples was isolated using Qiagen DNeasy kit (Qiagen, Hilden, Germany). *P. gingivalis* DNA was identified in host tumor tissue by RT PCR (BioRad, Haifa, Israel) using specific qPCR primers for the *P. gingivalis* 16S gene [6], forward sequence 5′-AGAGTTTGATCCTGCTCAG-3′ and reverse sequence 5′-CAATACTCGTATCGCCCGTTATTC-3′. The human 18S rRNA gene was used as an internal control for DNA extraction from the tumor tissue, forward sequence: 5′- CTACCACATCCAAGGAAGCA-3′, and reverse sequence: 5′- TTTTTCGTCACTACCTCCCCG -3′. The expression of *P. gingivalis* DNA in the tumor tissue relative to human DNA was calculated using the delta-delta Ct method (2^-ΔΔCt^).

### 4.9. Fluorescence In Situ Hybridization (FISH)

Excised tumor tissue was fixed overnight in 4% paraformaldehyde (Sigma) and embedded in Cryostat Embedding Medium (OCT, Fisher Scientific, Waltham, MA, USA), and frozen in liquid nitrogen. Frozen blocks were cryosectioned at 10 μm for FISH. Sections were washed in 99.5% ethanol and probed with 5 mg/mL of *P. gingivalis* 16s rRNA- specific oligonucleotide POGI 5′-CAA TAC TCG TAT CGC CCG TTA TTC-3′ (as per [31]) labeled with Cy3 dye (Hy-Labs, Rehovot, Israel) in hybridization solution (0.9 mM NaCl, 20 mM Tris/HCl, pH 7.3, and 0.01% SDS). Slides were incubated in a humid chamber at 46 °C for 3.5 h in the dark. Slides were then rinsed in sterile double distilled water, air dried, and counter stained with 1 μL/mL DAPI (Sigma-Aldrich). Slides were visualized using a NIKON eclipse 80i fluorescent microscope at 20× magnification.

### 4.10. Western Blot

Tumor tissue or *P. gingivalis* infected cells were lysed in RIPA lysis buffer containing protease and phosphatase inhibitors, and protein concentration was measured by Bradford (Bio-Rad, Hercules, CA, USA). Proteins were separated by SDS-PAGE and transferred to nitrocellulose membranes. Rabbit antibodies were from Cell Signaling (Cell Signaling, Danvers, MA, USA): anti-phospho-AKT (clone D9E), and anti-Akt (pan, clone C67E7). Primary rabbit antibodies were used at 2 µg/mL overnight, followed by secondary goat anti-rabbit IgG-HRP (Abcam, Cambridge, UK). ECL-chemiluminescence detection kit (Biological Industries) was used to detect the proteins, and images were captured using a Bio-Rad imaging system (Bio-Rad, Hercules, CA, USA). Total and phosphor-AKT levels were determined by densitometry. Detailed information about western blot can be found at Figures S5–S7 (Supplementary Materials).

### 4.11. Statistical Analysis

Significant differences between the means were analyzed using the unpaired Student’s *t* test or the two-way ANOVA (Prism v.8, GraphPad Software Inc., San Diego, CA, USA). A *p* value <0.05 was considered statistically significant.

## 5. Conclusions

Our findings, taken together with the previously published prospective epidemiologic studies, lead us to conclude that the periodontal bacterium *P. gingivalis* may act as a mechanistic determinant in pancreatic carcinoma progression. Moreover, our observations highlight the importance of the interplay between hypoxia (a characteristic feature of PDAC tumors) and *P. gingivalis* intracellular survival in pancreatic carcinoma cells.

## Supplementary Materials

The following are available online at https://www.mdpi.com/2072-6694/12/8/2331/s1, Figure S1: Pancreatic cancer cell proliferation induced by *P. gingivalis* is independent of TLR2, Figure S2: Increased expression of Cyclin-D1 and Heparanase in PANC-1 cells infected with *P. gingivalis*, Figure S3: *P. gingivalis* induces sustained phospho-Akt in PANC1 cells, Figure S4: *P. gingivalis* enhances Akt phosphorylation in MIA PaCa-2 cells, Figure S5: Detailed information about Figure 6d, Figure S6: Full length gels and densitometry readings for Figure S3, Figure S7: Full length gels and densitometry readings for Figure S4.
