# Supplementary material for: Intracellular Porphyromonas gingivalis Promotes the Tumorigenic Behavior of Pancreatic Carcinoma Cells
Source: Cancers (Basel). 2020 Aug 18;12(8):2331. doi: 10.3390/cancers12082331 (PMC7465784; doi:10.3390/cancers12082331)
Supplement: Supplementary file 1 [file cancers-12-02331-s001.pdf]

Supplementary Materials

Intracellular *Porphyromonas gingivalis* Promotes the Tumorigenic Behavior of Pancreatic Carcinoma Cells

JebaMercy Gnanasekaran [1], Adi Binder Gallimidi [1,2], Elias Saba [1], Karthikeyan Pandi [1], Luba Eli Berchoer [1], Esther Hermano [2], Sarah Angabo [1], Hasna'a Makkawi [1], Arin Khashan [1], Alaa Daoud [1], Michael Elkin [2,*,†] and Gabriel Nussbaum [1,*,†]

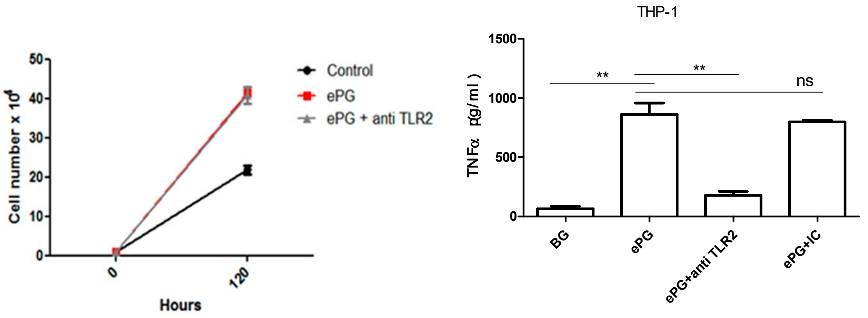

Figure S1. Pancreatic cancer cell proliferation induced by *P. gingivalis* is independent of TLR2. Enhanced PANC-1 proliferation induced by ePG is not inhibited by blocking TLR2 with the inhibitory T2.5 monoclonal antibody (mAb). As a control for the inhibitory activity of the T2.5 mAb, human THP-1 macrophages were treated with the T2.5 mAb vs. isotype control mAb (IC) and then stimulated with ePG overnight. Supernatants were collected and tested for TNFa production by ELISA (ns = nonsignificant, ** $p < 0.01$). The T2.5 mAb inhibits macrophage stimulation by *P. gingivalis*.

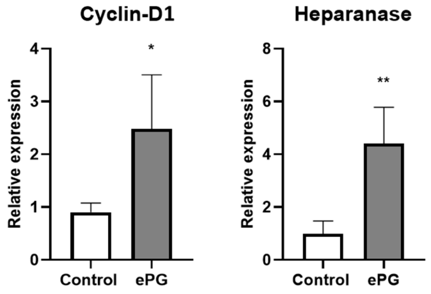

Figure S2. Increased expression of Cyclin-D1 and Heparanase in PANC-1 cells infected with *P. gingivalis.* PANC1 cells were grown in hypoxia, and cultured alone or infected in vitro with ePG for 24h as described in Methods. Quantitative RT-PCR was used to assess expression of Cyclin-D1 and Heparanase. * $p < 0.05$, ** $p < 0.01$.

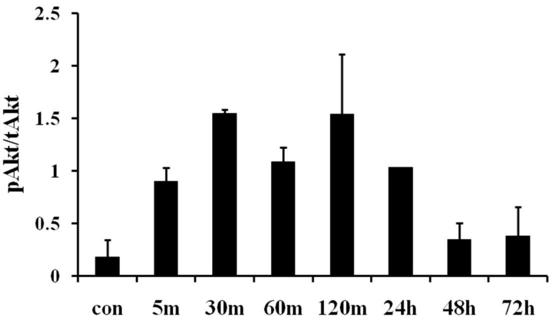

Figure S3. *P. gingivalis* induces sustained phospho-Akt in PANC1 cells. PANC1 cells were grown in hypoxia, infected in vitro with ePG for the times indicated, and then lysates were prepared and analyzed by western blot. Protein levels were determined by densitometry. The experiment was performed twice. Data show the mean ± SD of the combined results of the experiments.

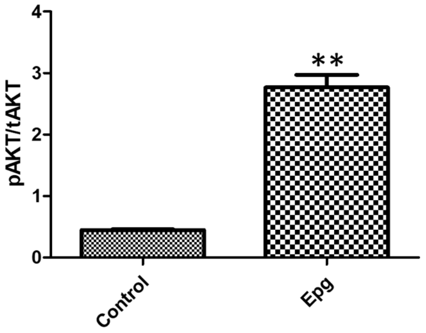

Figure S4. *P. gingivalis* enhances Akt phosphorylation in MIA PaCa-2 cells. MIA PaCa-2 cells were infected with ePG for 1h, treated with metronidazole and gentamicin for 1h, and then incubated for 24 h. All steps were performed in hypoxia. Cell lysates were analyzed by WB for phosphorylated and total Akt, and protein levels were determined by densitometry. The experiment was performed in duplicate. ** $p = 0.0074$.

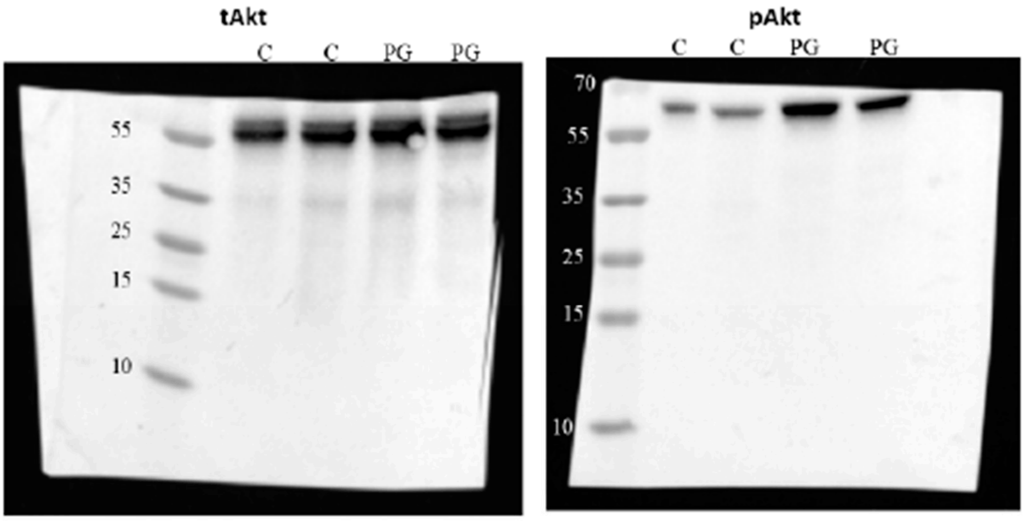

	pAkt	tAkt
Control	10024.05	25665.17
Control	13100.9	29155.56
PG	36470.97	31197.46
PG	28666.75	28115.13

Figure S5. Full length gels and densitometry readings for Figure 6d.

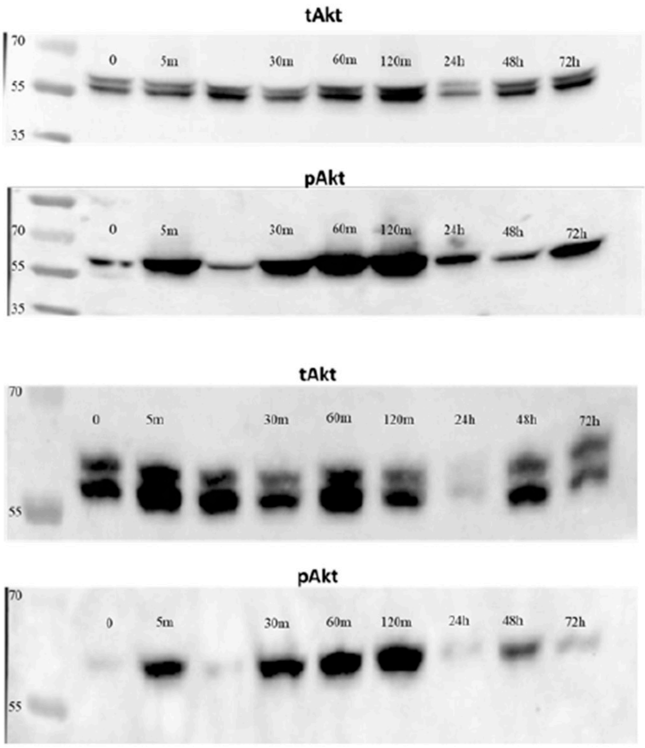

	pAkt	tAkt
con	2025.548	6321.891
5m	9149.711	9052.225
30m	8139.326	5379.69
60m	11511.08	9577.134
120m	14672.28	14025.5
24h	4897.761	4740.255
48h	2168.012	10092.67
72h	6462.983	10429.23

	pAkt	tAkt
con	425.335	9526.962
5m	10925.35	13807.01
30m	13253.76	8420.841
60m	13092.33	13550.67
120m	16644.86	8205.134
24h	1131.962	1096.184
48h	4933.255	10246.67
72h	1090.983	7692.134

Figure S6. Full length gels and densitometry readings for Figure S3. (Membranes were cut prior to processing.).

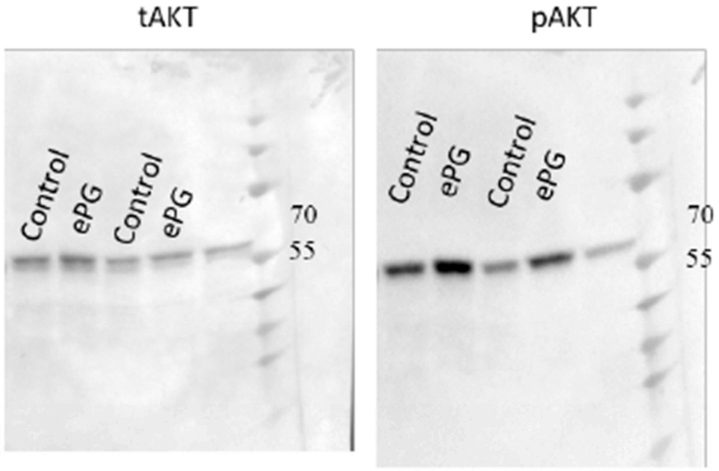

	pAKT	tAKT	ratio
Control	2464272	3155460	0.780955
ePG	9520488	3514275	2.70909
Control	1236620	1930225	0.640661
ePG	8288160	2702848	3.066454

Figure S7. Full length gels and densitometry readings for Figure S4.

© 2020 by the authors. Licensee MDPI, Basel, Switzerland. This article is an open access article distributed under the terms and conditions of the Creative Commons Attribution (CC BY) license (http://creativecommons.org/licenses/by/4.0/).
